# Hepatic Inflammation Confers Protective Immunity Against Liver Stages of Malaria Parasite

**DOI:** 10.3389/fimmu.2020.585502

**Published:** 2020-11-19

**Authors:** Morgane Grand, Mishelle Waqasi, Claudia Demarta-Gatsi, Yu Wei, Roger Peronet, Pierre-Henri Commere, Amandine Puig, Jonathan Axelrod, Reto Caldelari, Volker Heussler, Rogerio Amino, Salaheddine Mecheri

**Affiliations:** ^1^ Institut Pasteur, Unité de Biologie des Interactions Hôte Parasites, Paris, France; ^2^ CNRS ERL9195, Paris, France; ^3^ INSERM U1201, Paris, France; ^4^ Collège Doctoral, Sorbonne Université, Paris, France; ^5^ Institut Pasteur of Shanghai, Chinese Academy of Sciences, University of Chinese Academy of Sciences, CAS Key Laboratory of Molecular Virology and Immunology, Shanghai, China; ^6^ Institut Pasteur, Unité de Virologie Moléculaire et Vaccinologie, Paris, France; ^7^ Institut Pasteur, Imagopole, Paris, France; ^8^ Goldyne Savad Institute of Gene Therapy, Hadassah Medical Organization, Jerusalem, Israel; ^9^ Institute of Cell Biology, University of Bern, Bern, Switzerland; ^10^ Institut Pasteur, Malaria Infection and Immunity Unit, Paris, France

**Keywords:** CD8-CD4 T cells, inflammation, liver, malaria, vaccine

## Abstract

Deciphering the mechanisms by which *Plasmodium* parasites develop inside hepatocytes is an important step toward the understanding of malaria pathogenesis. We propose that the nature and the magnitude of the inflammatory response in the liver are key for the establishment of the infection. Here, we used mice deficient in the multidrug resistance-2 gene (Mdr2^−/−^)-encoded phospholipid flippase leading to the development of liver inflammation. Infection of Mdr2^−/−^ mice with *Plasmodium berghei* ANKA (*Pb*ANKA) sporozoites (SPZ) resulted in the blockade of hepatic exo-erythrocytic forms (EEFs) with no further development into blood stage parasites. Interestingly, cultured primary hepatocytes from mutant and wild-type mice are equally effective in supporting EEF development. The abortive infection resulted in a long-lasting immunity in Mdr2^−/−^ mice against infectious SPZ where neutrophils and IL-6 appear as key effector components along with CD8^+^ and CD4^+^ effector and central memory T cells. Inflammation-induced breakdown of liver tolerance promotes anti-parasite immunity and provides new approaches for the design of effective vaccines against malaria disease.

## Introduction

Malaria infection has two distinct phases in the mammalian host: a clinically silent pre-erythrocytic phase that is required for the establishment of the infection, and an erythrocytic phase, responsible for all the clinical symptoms of malaria. As the development of *Plasmodium* sporozoites (SPZ) inside hepatocytes is an essential step before the onset of disease, the liver is an obligate tissue which offers an ideal niche for transmitted SPZ to develop.

The liver is a body site where immunological tolerance mechanisms prevail (1). This property, which has evolved to limit over-reactions of this tissue to the continuous flow of antigens and pro-inflammatory stimuli originating from the intestine, represents an immunologic advantage to pathogens that have elected the liver as the site for their development (2, 3). Tolerance elicited *via* the portal vein (4) seems to be mediated by Kupffer cells (KC) (5) and was shown to be involved in tolerance to liver allografts, to tumor metastases, and to the persistence of hepatitis B and C viruses (6, 7). Cross-presentation of antigen *in vitro* by KC was shown to inhibit T cell activation in an IL-10-dependent manner (8) and to cause *in vivo* CD8 T cell apoptosis (9). In case of liver transplants, allografts are spontaneously accepted with only low-dose immune-suppression and induce tolerance for non-hepatic co-transplanted allografts of the same donor. Although this immuno-tolerogenic environment is favorable in the setting of organ transplantation, it is detrimental in chronic infectious liver diseases like hepatitis B or C, schistosomiasis, and malaria, or tumorigenesis, leading to pathogen persistence and weak anti-tumor effects (10). Other liver cells participate in tolerance mechanisms as well. Liver sinusoidal endothelial cells were demonstrated to promote CD8 T cell tolerance *via* decreased expression of co-stimulatory molecules CD80 and CD86 (11) and up-regulation of the checkpoint inhibitor PD-L1 (12). Hepatic stellate cells, located between hepatocytes and liver sinusoidal endothelial cells, have also been reported to be poor antigen presenting cells and tolerogenic through the expansion of regulatory T cells (13).

These findings suggest that during the establishment of *Plasmodium* infection, the invasion and development of infectious SPZ inside hepatocytes take advantage of the liver tolerogenic environment. Understanding the immunological mechanisms associated with the liver tissue is of paramount importance during malaria infection since it acts as a bottleneck to blood-stage infection and represents a potential site for parasite and disease control with the development of effective therapeutics and/or vaccines. Additionally, this tissue represents the only non-lymphoid organ where priming of CD8 and CD4 T cells can take place independently of lymphoid tissues (14). In fact, it was observed that the development of SPZ into the EEFs occurs in absence of any inflammatory reaction (15). Importantly, reversal of the tolerance state into inflammation was shown to result in an efficient adaptive immune response and anti-parasite protection, a condition that was observed during immunization with γ-irradiated SPZ (15).

The liver tolerance can be reversed under inflammatory conditions leading to the recruitment of leukocytes involved in the innate and adaptive immunity. The purpose of this study is to use a genetically engineered mouse model with a high inflammatory environment to investigate if shifting liver tissue homeostasis toward an inflammatory state would promote anti-parasite immunity. Mice deficient in the multidrug resistance-2 gene (Mdr2^−/−^), which encodes the canalicular phospholipid flippase leading to a complete absence of phospholipids from bile, spontaneously develop liver injury with features resembling human sclerosing cholangitis and low grade hepatitis (16). The bile of these mice does not contain phosphatidylcholine, the major phospholipid of the bile which serves to emulsify bile acids secreted by hepatocytes and thereby attenuates their toxicity. The Mdr2^−/−^ mice, which have been exploited in a pioneering study to demonstrate the relevance of inflammation for liver carcinogenesis (16, 17), provide here a genuine tool to explore the relationships between liver inflammatory processes and immunity against liver stage *Plasmodium* infection.

## Material and Methods

### Ethics Statement

All animal care and experiments described in the present study involving mice were conducted at the Institut Pasteur, approved by the “Comité d’Éthique en Expérimentation Animale” (CETEA) (Permit Number N° dap180040 issued on 2018) and performed in compliance with institutional guidelines and European regulations. A statement of compliance with the French Government’s ethical and animal experiment regulations was issued by the Ministère de l’Enseignement Supérieur et de la Recherche under the number 00218.01.

### Mice

Six- to eight-week-old female Mdr2 knock-out homozygous in FVB and C57BL/6JRj genetic backgrounds were used for this study, as well as the double knock-out Mdr2^−/−^ IL-6^−/−^ (C57BL/6JRj). The WT FVB and C57BL/6JRj mice were used as controls. Mdr2^−/−^ (FVB) were obtained from Yu Wei (Department de Virologie, Institut Pasteur) Mdr2^−/−^ (C57BL/6JRj) and Mdr2^−/−^ IL-6^−/−^ (C57BL/6JRj) were obtained from Jonathan Axelrod (Goldyne Savad Institute of Gene Therapy, Hadassah Medical Center, Israel) (18). WT FVB mice were purchased from Charles River Laboratories and C57BL/6JRj mice were purchased from Janvier Labs, France. Colonies of both Mdr2^−/−^ (FVB), Mdr2^−/−^ (C57BL/6JRj), Mdr2^−/−^IL-6^−/−^ (C57BL/6JRj) and WT FVB mouse strains were maintained at the animal facility of Institut Pasteur. FVB is an albino, inbred laboratory mouse strain that is named after its susceptibility to Friend leukemia virus B. FVB/NJ are commonly used for transgenic injection and have the *H2^q^* MHC haplotype. Animals were housed with a 12-hour light-dark cycle with standard chow and water.

### Parasites and Mice Infection

Mice were infected with either *Pb*NK65 or *Pb*ANKA GFP-transgenic SPZ collected from salivary glands of infected *Anopheles stephensi*. Infections were performed *via* i.v. injection in the tail vein of either 10^2^, 10^3^, 10^4^ or 5 × 10^4^ SPZ according to the experiments or by natural infection by infectious mosquito bites. The natural infection was performed by the exposure of mice to bites of 10 infected *A. stephensi* for 15 min. Mosquitoes were provided by the CEPIA (Centre d’élevage, de production et d’infection des anopheles, Institut Pasteur). Survival and parasitemia as determined by FACS using Cytoflex cytometer (Beckman Coulter Life Sciences, Villepinte, France) and the software FlowJo (FlowJo LLC, Ashland, OR, USA) were then monitored daily, beginning day 4 p.i. Symptoms associated with ECM in mouse models include coat ruffling, a respiratory distress syndrome, a drop in body temperature, and neurological signs such as paralysis, and coma, followed by death. For ethical reasons, manifestation of signs such as coat ruffling and reduced motor skills which represent a limit point, constitutes a criterion for interrupting the experience.

### Preparation of Mouse Liver RNA for Quantification of Parasite Load and Cytokines

The livers of Mdr2^−/−^ and WT mice infected with 10^4^ SPZ of *Pb*ANKA were surgically removed 4, 12, 24 and 40 h p.i. respectively. Total RNAs were extracted from the liver samples using the guanidinium–thiocyanate–phenol–chloroform method (all from Invitrogen, Waltham, MA, USA). RNA was thereafter reverse transcribed by PCR (temperature: 65°C for 5 min, 42°C for 50 min, 70°C for 15 min) using 100 U SuperScriptTM II reverse transcriptase (RT) (Invitrogen, Waltham, MA, USA), 40 U RNAse Inhibitor and 2 μM oligo(dT) per sample. The expression levels of diverse parasite and mouse transcripts were analyzed by real time RT-qPCR using Power SYBR^®^ Green PCR Master Mix (Applied Biosystems Foster City, CA, USA) and various primer sets (**Table S1**). All reactions were performed in the CFX96 Touch™ Real-Time PCR Detection System (Bio-Rad Laboratories Inc., Hercules, CA, USA) (temperature: 50°C for 2 min, 95°C for 10 min, 40 cycles of 95°C for 15 s and 60°C for 1 min). The relative abundance of the target RNAs in the liver was calculated using the ΔCt method, and expressed as (2^−ΔCt^) × 10^4^. The mouse hypoxanthine phosphoribosyl transferase (HPRT) gene was used as an internal control for the variation in input RNA amounts. A no template control (NTC) was included to ensure that there was no cross-contamination during sample preparation. The same protocol was used for RNA extraction from isolated neutrophils and hepatocytes.

### *Ex Vivo* Microscopy for Quantification of EEF in Mice Liver

The livers of Mdr2^−/−^ and WT mice were removed 4, 12, 24 and 40 h respectively after being injected with 50,000 GFP-expressing *Pb*ANKA SPZ. Livers were placed in PBS in a 35 mm imaging dish with Polymer Coverslip Bottom (Ibidi GmbH, Gräfelfing, Bayern, Germany) and observed using a Zeiss Axiovert (Zeiss, Oberkochen, Germany) equipped with phase-contrast and epifluorescence microscopy. EEF were counted on 20 fields (objective × 10) and normalized by 10 mm^2^. Parasite size was determined on microscopy pictures using the ImageJ software.

### Culture and Infection of Mouse Primary Hepatocytes

The livers of anesthetized mice, injected with a solution of Ketamine/Xylazine (Imalgene 1000/Rompun 2%), were perfused *via* the portal vein with two solutions. First 50 ml of 37°C Hepes buffer (137 mM NaCl, 2.68 mM KCl, 10 mM Hepes, 0.7 mM Na2HPO4) complemented with penicillin/streptomycin (100 U/ml). Then with 50 ml of 37°C William’s E medium (Lonza Group GmbH, Waldshut-Tiengen, Germany) containing 100 U/ml of type IV collagenase (Worthington Biochemical Corporation., Lakewood, NJ, USA). The perfusate was drained out of the body *via* the inferior vena cava, which was cut immediately after starting perfusion. During both perfusion periods, the inferior vena cava was clamped to inflate the liver and the perfusion solution was warmed at 37°C using a water bath. The digested liver was then excised rapidly, the liver capsule was disrupted, and the hepatocytes were released by gently shaking the digested liver into complete William’s medium E (10% FCS, 2 mM of L-Glutamin, 100 U/ml of Penicillin/Streptomycin, 1 μg/ml of Aprotinin, 10 μg/ml of human Transferrin, 1 μg/ml of human recombinant Insulin) (Sigma Aldrich, Saint-Louis, MO, USA). The cell suspension was passed through a 100 µl nylon cell strainer (Falcon^®^ Thermo Fisher Scientific Inc, Brebière, France) and washed and centrifuged three times at 50 g in complete William’s E medium. The cells were resuspended, and viability was checked by using the trypan blue exclusion method. An enrichment in living hepatocytes was performed using a Percoll gradient: 5 ml of an 80% isotonic Percoll solution (in PBS 1X) was applied at the bottom of a 14 ml Falcon and cells were resuspended in 5 ml of 40% isotonic Percoll solution (in PBS 1×) and gently applied over the top of the tube. The cell suspension was fractioned by centrifugation at 750*g* during 20 min without brake at 20°C. After Percoll gradient centrifugation, the live hepatocytes were collected at the border between the two layers. Cells were washed two times in complete William’s Medium and counted. For microscopy pictures (**Figure 3**) 60,000 cells were plated on µ-Slide 8 well plates (Ibidi GmbH, Gräfelfing, Bayern, Germany). For co-culture assays between 15,000 and 20,000 cells were seeded onto a 96-well plate (TPP Techno Plastic Products AG, Trasadingen, Switzerland) in complete William’s E medium. The growth medium was replaced 3 to 5 h after isolation. The cells were cultured at 37°C and 5% CO_2_. *Pb*ANKA GFP expressing parasites were obtained by dissecting salivary glands from infected female *A. stephensi* mosquito SPZ suspension in complete William’s E medium was added at a ratio of SPZ/hepatocytes of 1/4 for each well 24 h after plating. Parasite liver stage development was followed and quantified by counting the whole well by fluorescent microscopy using a Zeiss Axiovert (Zeiss, Oberkochen, Germany) microscope equipped with phase-contrast and epifluorescence.

### Flow Cytometric Analysis of Liver Leukocytes

Liver resident leukocytes were obtained from naïve and infected Mdr2^−/−^ and WT mice 40 h p.i. Briefly, mice were lethally anesthetized with a solution of Ketamine/Xylazine and mice livers were perfused with 30 ml of PBS 1× to remove red blood cells and circulating leukocytes. After the perfusion livers were digested *in vitro* in a collagenase D solution (0.05%) (Roche Molecular Systems Inc., Branchburg, USA) at 37°C for 45 min and mixed through a 70 μm cell strainer (Falcon^®^ Thermo Fisher Scientific Inc, Brebière, France). Cells were washed in PBS and purified by Percoll gradient: 5 ml of an 80% isotonic Percoll solution (in PBS 1×) was applied at the bottom of a 14 ml Falcon and cells were resuspended in 5 ml of 40% isotonic Percoll solution (in PBS 1×) and gently applied over the top of the tube. The cell suspension was fractioned by centrifugation at 3,000 rpm during 30 min without brake at 4°C. After Percoll gradient centrifugation, the leukocytes were collected at the border between the two layers. Cells were washed in cold PBS, resuspended in FACS buffer containing 2% Fetal calf serum (FCS) and 0.01% sodium azide and counted. Obtained liver leukocytes were stained for FACS analysis according to standard protocols in a FACS buffer with the following antibodies: FITC-labeled CD4 (clone H129.19), PerCP-labeled anti-CD8 (clone (53–6.7), PerCP-labeled anti-F4/80 (clone BM8), FITC-labeled ant-Ly6G (clone 1A8), FITC-labeled anti-B220 (clone RA3-6B2), PE-labeled anti-CD11c (clone HL3), PerCP-labeled anti-CD19, APC-Cy 7 labeled anti-CD3 (clone 145-2C11), PeCy7-labeled anti-CD44 (clone IM7), PE-labeled anti-CD69 (clone H1 2F3), BV605 labeled anti-CD62L (clone MEL-14), BV421 hamster anti-mouse KLRG1 (clone 2F1) and FITC/PE/PerCP or Alexa fluor 647-labeled anti-CD45 (clone 30-F11). All antibodies were purchased from BD Biosciences, San Jose, CA, USA. Cells were washed and resuspended in FACS buffer before analysis. A total of 5 × 10^5^ living or fixed cells were analyzed using a Cytoflex cytometer (Beckman Coulter Life Sciences, Villepinte, France) and the software FlowJo (FlowJo LLC, Ashland, OR, USA).

### *In Vitro* Co-Cultures

WT FVB primary hepatocytes were isolated as described above and seeded in a 96-well plate, between 15,000 and 20,000 cells per well in complete William’s E medium. HepG2 cell line, routinely tested negative for mycoplasma (PlasmoTest, InvivoGen, San Diego, CA), were thawed in complete DMEM medium containing 10% FCS and 2% Streptomycin/Neomycin and plated at a concentration of 4 × 10^4^ cells per well. The day after plating, neutrophils were isolated from the bone marrow of Mdr2^−/−^ or control mice using magnetic beads based on negative selection using the Neutrophil Isolation kit (MACS, Miltenyi Biotec GmbH Bergisch Gladbach, Germany). Resident liver leukocytes were isolated from Mdr2^−/−^ and WT FVB mice as described previously. Both liver leukocytes or neutrophils were added to hepatocyte cultures at different ratios leukocyte/hepatocytes (1/10, 1/100 and 1/1,000) 2 h before the addition of SPZ into the cultures. Parasite liver stage development was followed and quantified by counting the whole well by fluorescent microscopy using a Zeiss Axiovert microscope equipped with phase-contrast and epifluorescence.

### *In Vivo* Neutrophil Depletion

For neutrophil depletion, Mdr2^−/−^ mice were injected with 250 μg of a rat anti-mouse neutrophil depleting antibody (anti Ly6G rat monoclonal antibody, clone Nimp-R14 which was shown to recognize the same neutrophil population as the anti-Ly6B 7/4 mAbs.) provided by G. Milon (Institut Pasteur) the day before the infection with 10^4^ GPF-expressing *Pb*ANKA. Control mice were injected with an unrelated IgG2b isotype control. The depletion was assessed by flow cytometry, on blood samples collected from the tail vein of treated and untreated mice. Neutrophils were counter-stained using an FITC-conjugated anti-neutrophil 7/4 antibody which recognizes Ly6GB surface antigen. In addition, as neutrophils are the major side scatter-high (SSC^high^) leukocyte population, treatment with a neutrophil-depleting antibody induces marked reduction in the number of SSC^high^ cells.

### Treatment of Mice With Murine Recombinant IL-6

The murine recombinant IL-6 (ImmunoTools GmbH, Friesoythe, Germany) was administered i.v. at various doses, 1, 5, and 10 μg/mouse to WT FVB mice four times: 1 day before, 1 h before, 1 day, and 2 days after infection with *Pb*ANKA SPZ. Control mice were injected with PBS.

### Detection of Anti-Parasite Specific IgG Antibodies

To detect parasite-specific antibodies, 96-well plates (Nunc-immuno plate; Thermo Scientific, Rockford, IL) were coated with parasite protein extract from isolated SPZ from *A. stephensi* salivary glands in carbonate buffer, pH 9.6, for 2 h at 37 °C. After the plates were saturated with 1% (w/v) pork gelatine, each serum sample was assayed at serial dilutions and incubated overnight for 2 h at 37°C. Specific binding was detected using horseradish peroxydase (HRP)-conjugated goat anti-mouse secondary antibody (Cell Signaling technology^®^, Danvers, MA) followed by the addition of o-phenylenediamine dihydrochloride (OPD) substrate (Sigma-Aldrich; St. Louis, MO). Hydrogen chloride (HCl) 1 N was used to block the reaction. The optical density (OD) was read at 490–655 nm. Each sample was tested against non-immune serum and PBS as background controls.

### Statistical Analysis

All data were analyzed using Prism 5.0 software (GraphPad Software, San Diego, USA). Unpaired data between two groups at a specific time point were analyzed by a Mann–Whitney test for nonparametric analysis. Kaplan–Meier survival plots were analyzed using a Mantel–Cox test. A p-value <0.05 was considered to be statistically significant. All experiments were replicated several times as indicated in the figure legends.

## Results

### Parasite Development Is Blocked in the Liver of Mdr2^−/−^ Mice

**Figure 1:**
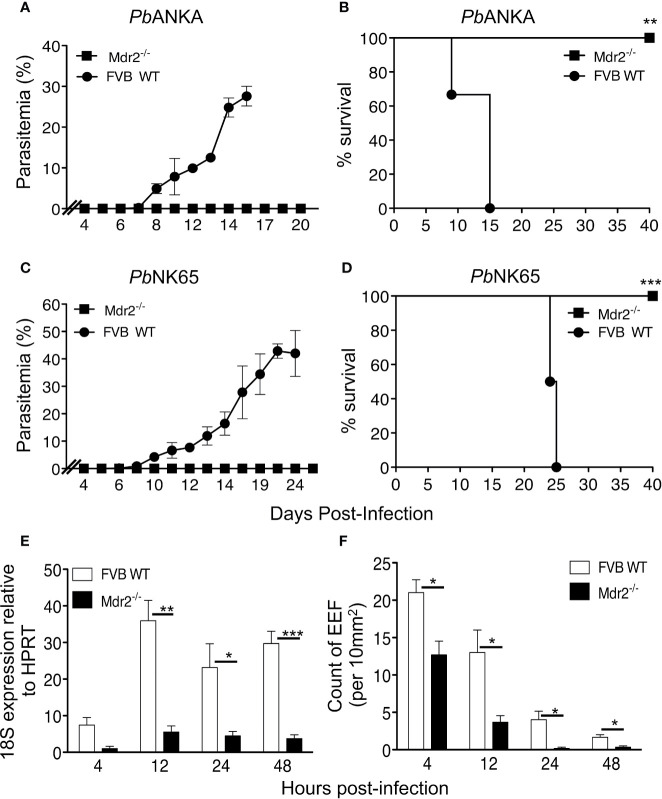
Mdr2^−/−^ mice do not develop blood stage parasites. Parasitemia and survival of infected WT FVB and Mdr2**^−/−^** mice. Six-week-old WT FVB and Mdr2**^−/−^** mice (n = 6 per group) were naturally infected by the exposure to bites of 10 Anopheles infected with *Pb*ANKA **(A, C)** or *Pb*NK65 **(B, D)**. Survival rate (Kaplan–Meier survival rates, Mantel–Cox test **p = 0.0012; ***p = 0.0009) and parasitemia were recorded over time. Results are from two independent experiments. **(E)** Real time qPCR (RT-qPCR) quantification of parasite ribosomal RNA 18s into 6-week-old female WT FVB and Mdr2^-/-^ mice 4, 12, 24 and 40 h after infection with 10^4^ SPZ (n = 5 per group). RT-qPCR was performed using standard SyberGreen protocols on total liver RNA. **(F)** Quantification by *ex vivo* microscopy of exoerythrocytic forms (EEFs) in livers of 6-week-old female WT FVB and Mdr2**^−/−^** mice injected i.v. with 5 × 10^4^ *Pb*ANKA SPZ 4, 12, 24 and 40 h post infection (n = 5 per group). EEFs were counted on 20 fields (objective × 10) and normalized by 10 mm^2^ of liver section. Results are from two independent experiments (Mann–Whitney test; *p < 0.05; **p < 0.01; ***p < 0.001).

Mice were exposed to infectious mosquito bites delivering either *Pb*ANKA or *Pb*NK65, two lethal strains of parasites, which cause experimental cerebral malaria (ECM) (19) or hyperparasitemia and anemia (20, 21), respectively. **Figures 1A, B** show that in contrast to WT mice, Mdr2^−/−^ mice did not develop blood stage infections with both parasites. All WT mice infected with both parasites died, whereas all Mdr2^−/−^ mice survived the infection (**Figures 1C, D** **)**.

To control the effect of the actual amount of inoculated SPZ, infection with various doses of isolated parasites (10^2^, 10^3^ and 10^4^ SPZ, respectively) from the mosquito salivary glands showed that in contrast to WT FVB mice, infection of Mdr2^−/−^ mice with *Pb*ANKA SPZ at all doses, except at the highest dose where a transient parasitemia was noticed (**Supplementary Results: Figure 1E**), resulted in a complete blockade of the parasite in the liver (**Supplementary Results: Figures 1A, C, E**). The survival curves are in agreement with those of parasitemia and none of the Mdr2^−/−^ mice died from ECM except one out of six which died from ECM at the dose of 10^4^ SPZ (**Supplementary Results: Figures 1B, D, F**). In comparison, all WT FVB mice infected with 10^2^ SPZ and half of the mice which received the dose of 10^3^ SPZ died from hyperparasitemia but not CM, whereas the other half of the mice which were infected with 10^3^ SPZ and 80% of the mice which received the dose of 10^4^ SPZ died from CM (**Supplementary Results: Figures 1B, D, F**). Similarly, inoculation of Mdr2^−/−^ mice with *Pb*NK65 at the same range of doses did not result in any infection and all mice survived the infection (**Supplementary Results: Figures 1G, I, K**), in contrast to WT mice which, depending on the dose of the inoculum, developed parasitemia and at the highest dose of 10^4^ SPZ all mice developed parasitemia and ultimately died from hyperparasitemia and anemia (**Supplementary Results: Figures 1H, J, L**).

**Figure 2:**
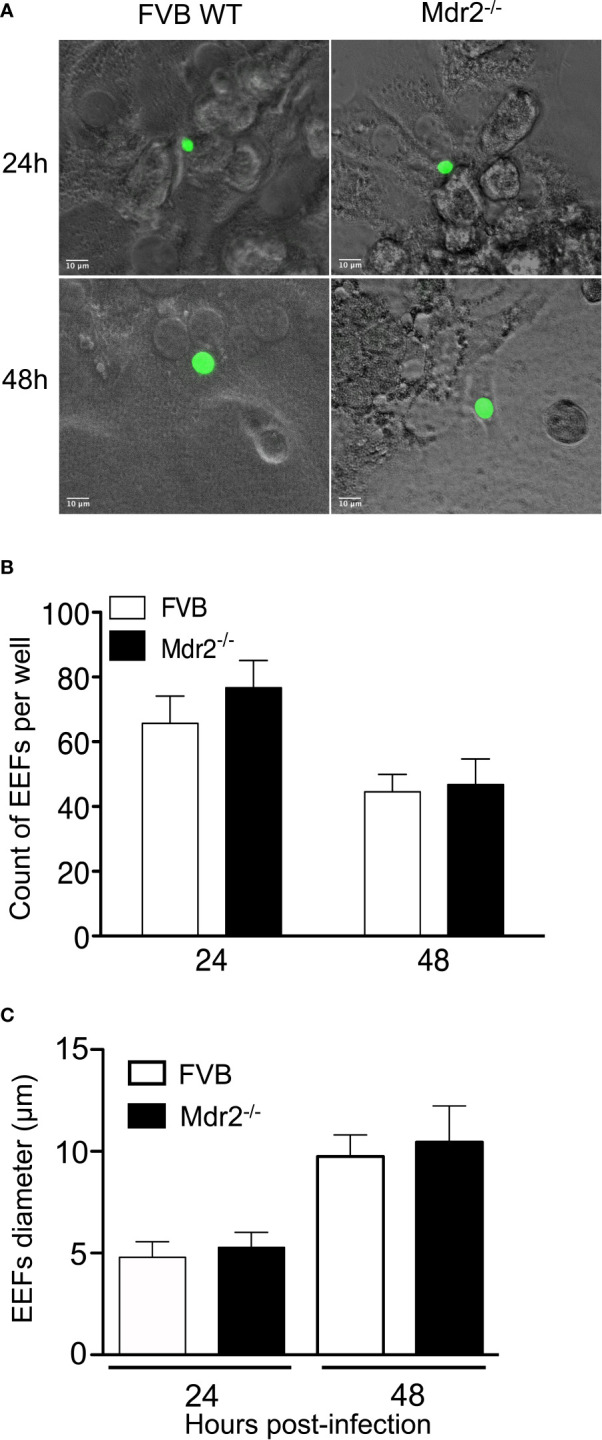
Isolated primary hepatocytes from WT and Mdr2^−/−^ mice are equally able to support EEF development.*** ***Hepatocytes were isolated from 6-week-old female mice by a two steps perfusion of the entire liver *in vivo* with collagenase IV and infected *in vitro* with *Pb*ANKA SPZ. **(A)** Representative EEFs (in green) shown by fluorescence microscopy 24 and 48 h p.i. of *in vitro* infected hepatocytes from WT FVB and Mdr2**^−/−^** mice by fluorescence microscopy (Objective × 40). **(B)** Quantitative representation of EEFs counted by fluorescence microscopy on 20 fields (objective × 10). Counts were performed on eight wells per condition (Mann Whitney test; ns p > 0.05). **(C)** Quantitative representation of EEFs size. Diameters of parasites were measured using Image J software (n = 6 per group) (Mann Whitney test; ns p > 0.05). Bar graphs represent the mean ± SEM. Experiments were replicated three times.

To the best of our knowledge, no previous reports exist on infections of Mdr2^−/−^ mice with *P. berghei* parasites. Here, we provide evidence that, in contrast to SPZ infection, these mice support perfectly well *Pb*ANKA growth when inoculated with blood stage parasites, indicating that the liver constitutes a true barrier for SPZ development. This is clearly shown in **Supplementary Results: Figure 2** where both parasitemia (**Supplementary Results: Figure 2A**), except at days 13–15 where some differences were observed, and survival rates (**Supplementary Results: Figure 2B**) where indistinguishable between WT FVB and Mdr2^−/−^ mice. It can also be inferred that the inflammatory environment in these animals is not simply suppressive of asexual parasite growth but rather represents a failure to complete liver stage development.

These data prompted us to quantitatively assess the parasite load in the liver. Female WT and Mdr2^−/−^ mice were inoculated intravenously (iv) with 10^4^ SPZ and liver samples collected at 4, 12, 24, and 48 h post-infection (p.i) were subjected to qRT-PCR analysis of parasite 18S rRNA. While in WT mice the presence of the parasite was detected at 4 h and then progressed afterwards through 48 h p.i, the parasite was just present at the limit of detection throughout the entire pre-erythrocytic phase in Mdr2^−/−^ mice (**Figure 1E**). In parallel, we examined the abundance of exoerythrocytic forms (EEFs) in the liver of WT and Mdr2^−/−^ mice inoculated with 5 × 10^4^ *Pb*ANKA SPZ by fluorescence microscopy at the same time points as in **Figure 1E**. As shown in **Figure 1F**, EEFs were readily observed at 4h p.i. and decreased progressively over time in WT mice. Only about half number of EEFs were detected in Mdr2^−/−^ mice at 4 h p.i, when compared to WT mice, and then drastically decreased up to 48 h p.i. Altogether, these results suggest that parasites are blocked in the liver of Mdr2^−/−^ mice at early time points right after infection, and that clearance of SPZ and liver stages are both likely to be targeted by immune effector mechanisms.

### Isolated Primary Hepatocytes From WT and Mdr2^−/−^ Mice Are Equally Able to Support EEF Development

Previous *in vivo* results showed an impairment of parasite development in the livers of Mdr2^−/−^ mice. To assess whether hepatocytes from WT and Mdr2^−/−^ mice display a differential susceptibility to infection by SPZ, primary hepatocytes from WT and Mdr2^−/−^ mice were isolated and infected with *Pb*ANKA SPZ *in vitro*. As shown in **Figure 2**, *Pb* parasites can invade and develop inside primary hepatocytes isolated from WT and Mdr2^−/−^ hepatocytes with the same efficiency both in terms of parasite size (**Figure 2A**) or parasite counts (**Figure 2B**). The Mdr2^−/−^ mutation does not affect the ability of hepatocytes to support EEF development. These results suggest that the parasite development failure in the liver of Mdr2^−/−^ mice is more likely due to an active induction of immunity rather than to the inability of mutant hepatocytes to get infected.

### Cellular Infiltrates in Naïve and Infected Mdr2^−/−^ Mice

**Figure 3:**
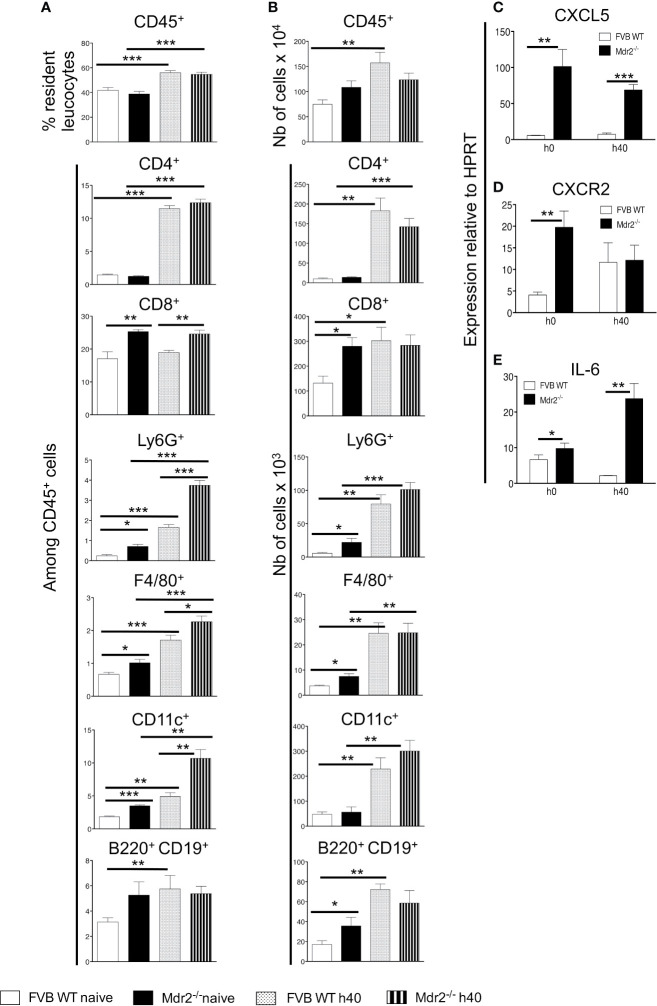
Patterns of resident liver leukocytes are differentially regulated in Mdr2^−/−^ mice. Livers of 6-week-old female WT FVB and Mdr2**^−/−^** mice were harvested from naive mice and 40 h p.i. with 10^4^ *Pb*ANKA SPZ (n = 6 per group). Leukocytes present in liver tissue were analyzed using the following markers: CD45, CD4, CD8, Ly6G, F4/80, CD11c, B220 and CD19. Results are expressed in percentage of positive cells among CD45^+^ cells **(A)** or in absolute cell number **(B)**. Results are representative for three independent experiments (Mann–Whitney test; *p < 0.05; **p < 0.01; ***p < 0.001). **(C)** Neutrophil chemoattractant chemokines and chemokine receptors and IL-6 are up-regulated in Mdr2**^−/−^** mouse livers. Quantitative analysis by RT-qPCR of CXCL5 **(B)**, CXCR2 **(D)** and IL-6 **(E)** on total liver mRNA extracted from 6-week-old female WT FVB and Mdr2**^−/−^** uninfected or 40 h p.i. with 10^4^ *Pb*ANKA SPZ (n = 6 per group). Results are representative of three independent experiments (Mann–Whitney test; *p < 0.05; **p < 0.01; ***p < 0.001).

To examine the changes in cellular infiltrates that may be caused by the *mdr2* mutation, we carried out a cell phenotyping analysis on perfused liver samples collected from naïve mice or 40 h p.i from mice inoculated i.v with 10^4^ SPZ. The most notable results showed, except for the CD45^+^ cell population, elevated percentage of CD8^+^, Ly6G^+^, F4/80^+^, and CD11c^+^ in naïve Mdr2^−/−^ mice as compared to WT mice (**Figure 3A**). Of note, these differences persisted in SPZ-infected mice with higher proportions in mutant mice. In naïve mice, total cell counts follow the same pattern in that the numbers of CD8^+^, Ly6G^+^, F4/80^+^, CD11C^+^, and B220^+^ CD19^+^ cells were found to be more elevated in Mdr2^−/−^ mice as compared to WT mice (**Figure 3B**). However, in infected mice, these differences were wiped out. These data suggest that the rapid blockade of SPZ development in the liver of Mdr2^−/−^ mice is very likely due to the higher prevalence of innate and adaptive immune cells which may act coordinately to arrest parasite developmental progression.

As neutrophils are among innate immune cells that represent a first line of defense against pathogens, one of the candidates which guide neutrophil recruitment during immune responses, is CXCL5, a powerful attractant for neutrophils which acts *via* CXCR2 receptor (22, 23). As shown in **Figure 3C**, higher levels of CXCL5 transcripts were observed in Mdr2^−/−^ mice as compared to WT FVB mice both in naïve mice and at 40 h p.i. CXCR2 was differentially expressed only in naïve mice and became similarly expressed by the two mouse strains at 40 h p.i. (**Figure 3D**). In addition, among a large panel of cytokines measured, only IL-6 levels were also found to be highly expressed in Mdr2^−/−^ mice as compared to WT FVB mice, both at the basal level and 40 h p.i. (**Figure 3E**). These findings strongly suggest that IL-6 production was associated with a higher prevalence of neutrophils in Mdr2^−/−^ mice and may act in tandem to clear parasites.

### Intra-Hepatocyte Parasite Development in Mdr2^−/−^ Mice Is Impaired by Neutrophils

**Figure 4:**
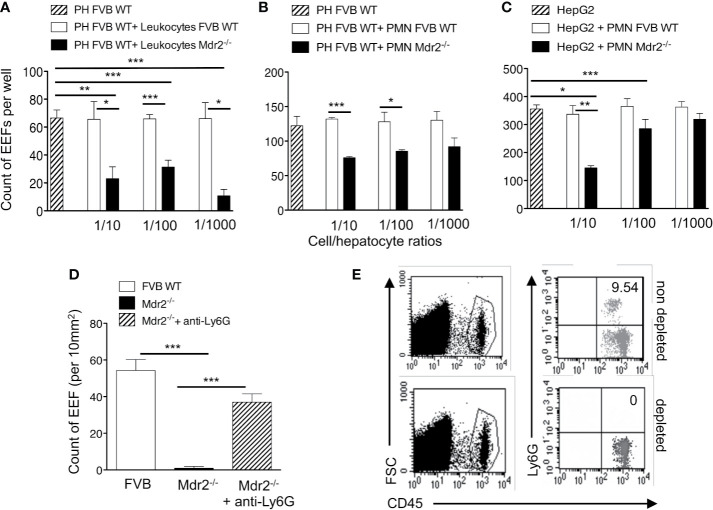
Impairement by neutrophils of intra-hepatic parasite development in Mdr2^-/-^ mice. Infection of primary hepatocytes (PH) isolated from naïve 6-week-old female WT FVB mice co-cultured with liver resident leukocytes **(A)** or with neutrophils isolated from the bone marrow of naïve 6-week-old female WT FVB and Mdr2^-/-^ mice **(B)**. In parallel, Infected HepG2 cell line was co-cultured with bone marrow neutrophils isolated from naïve 6-week-old female WT FVB and Mdr2^-/-^ mice. 1/10, 1/100 and 1/1000 reflect the ratios of leucocytes over hepatocytes **(A)** and neutrophils (PMN) over hepatocytes **(B, C)**. The number of EEFs was determined in five independent wells per condition using fluorescence microscopy (objective × 20) at 40 h p.i. with *Pb*ANKA. Results are representative of three independent experiments. **(D)** Measurement of EEFs by fluorescence microscopy (objective × 10) in the liver of WT FVB, and Mdr2**^−/−^** controls and in neutrophil depleted-Mdr2**^−/−^** mice 24 h p.i. with 10^4^ *Pb*ANKA SPZ (Mann–Whitney test; *p < 0.05; **p < 0.01; ***p < 0.001). **(E)** Assessment of neutrophil depletion efficacy: FACS dot plot analysis in the blood of 6-week-old female Mdr2**^−/−^** mice treated with the neutrophil depleting antibody anti-Ly6G or with an irrelevant antibody. Neutrophils are stained using CD45 and Ly6G markers. PH, primary hepatocyte; PMN, polymorphonuclear cells.

To test whether leukocytes interfere with infection of hepatocytes, resident leukocytes in Mdr2^−/−^ livers were isolated and co-cultured with primary hepatocytes isolated from WT FVB control mice at different ratios, two hours before infection with *Pb*ANKA SPZ. WT FVB hepatocytes alone or co-cultured with liver resident leukocytes from the same mouse strain were used as controls. Interestingly, the infection of WT FVB hepatocytes co-cultured with Mdr2^−/−^ leukocytes, as reflected by EEF counts at 40 h p.i., was significantly less efficiently infected than the infection in the controls whatever the leukocyte ratios (**Figure 4A**). This was reflected by a reduction by 87% of EEF counts at a Mdr2^−/−^ leukocyte/FVB hepatocyte ratio of 1 to 1,000. These data suggest that liver leukocytes of Mdr2^−/−^ mice interfere with the infection of primary WT hepatocytes *in vitro*.

In order to explore further the specific contribution of innate immune cell sub-populations in the decrease of infection efficiency by Mdr2^−/−^ mouse leukocytes, co-cultures were performed using neutrophils, a cell population found to be more prevalent in the livers of Mdr2^−/−^ mice (**Figure 3B**). Interestingly, we observed a significant decrease of EEF counts when *ex vivo* primary WT FVB hepatocytes were co-cultured with neutrophils isolated from Mdr2^−/−^ mice, but not with those from WT FVB mice (**Figure 4B**). Furthermore, a significant decrease of EEF counts was observed when HepG2 cells were co-cultured with neutrophils isolated from Mdr2^−/−^ mice at an Mdr2^−/−^ leukocyte/HepG2 hepatoma ratio of 1 to 10 (**Figure 4C**). Comparatively, Mdr2^−/−^ mouse neutrophils inhibited more effectively the infection of primary hepatocytes than of HepG2 cells. The ability of neutrophils, isolated from Mdr2^−/−^ mice, to dampen the infection *in vitro* suggests that these cells are key players in the developmental defect of the parasite in this model.

To address the relevance of neutrophils in the rapid clearance of *Plasmodium* parasites in the liver *in vivo*, Mdr2^−/−^ mice were treated with an anti-neutrophil depleting antibody before they were infected with 10^4^ *Pb*ANKA SPZ. As shown in **Figure 4D**, measurement of parasite load in the liver at 24 h p.i indicated a reversal in the development of the parasite in neutrophil-depleted mice reaching almost similar levels as in WT FVB mice as compared to undetectable parasites in the control untreated Mdr2^−/−^ mice. Efficacy of neutrophil depletion was assessed in the blood of treated mice by flow cytometry and showed a complete absence of neutrophils as compared to untreated mice (**Figure 4E**). These data provide evidence that neutrophils are key players in blocking EEF development *in vivo* in infected Mdr2^−/−^ mice.

### Reversal of Mdr2^−/−^ Associated Infection Phenotype in IL-6-Deficient Mice

**Figure 5:**
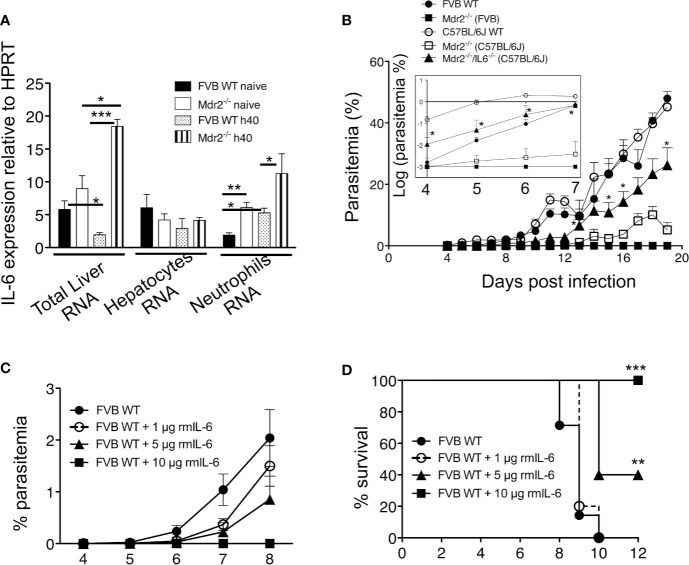
IL-6 is essential for the clearance of the parasite in Mdr2^−/−^ and is sufficient to abrogate parasite development in WT mice. **(A)** Quantification of IL-6 in total liver mRNA by RT-qPCR in isolated primary hepatocytes and in sorted bone marrow neutrophils of 6-week-old female WT FVB and Mdr2**^−/−^** (FVB) mice naive and infected with 10^4^ *Pb*ANKA SPZ (Mann–Whitney test; *p < 0.05; **p < 0.01; ***p < 0.001). **(B)** Parasitemia of infected 6-week-old female WT FVB, C57BL/6, Mdr2**^−/−^** (FVB), Mdr2**^−/−^** (C57BL/6) and Mdr2**^−/−^**IL-6**^−/−^** (C57BL/6) mice inoculated with 10^4^ *Pb*ANKA SPZ (n = 6 per group) were recorded over time. To highlight the subtle differences in parasitemia between different groups of mice, the insert represents the parasitemia between days 4 and 7 p.i. expressed in log scale. The asterisks indicate that significant differences exist between Mdr2**^−/−^**IL-6**^−/−^** (C57BL/6) and Mdr2**^−/−^** (C57BL/6) groups using the Mann–Whitney test (*p < 0.05). **(C, D)** Parasitemia and survival of 6-week-old female WT FVB mice (n = 6 per group) treated with several doses (1, 5, and 10 μg/mouse) of murine recombinant IL-6 (rmIL-6) and infected with 10^4^ *Pb*ANKA SPZ. Control mice were injected with PBS. **(D)** Survival rate was recorded over time (Kaplan–Meier survival plot, Mantel–Cox test **p < 0.0061, ***p = 0.001). Results are from two independent experiments.

At this point, it is tempting to postulate from the combined data obtained in **Figures 3** and **4** that neutrophils may act in concert with IL-6 to ensure parasite clearance in the liver of Mdr2^−/−^ mice. We first assessed IL-6 expression in the liver before and 40h after SPZ inoculation. As shown in **Figure 5A**, basal levels tend to be higher in total liver tissue of Mdr2^−/−^ mice and while IL-6 was significantly down-regulated in infected WT FVB mice, there was a dramatic up-regulation of IL-6 in the liver of infected Mdr2^−/−^ mice. This indicates that down-regulation of IL-6 by WT parasites which we observed before (24) was buffered and reversed upon the *mdr2* gene deletion and the pro-inflammatory environment that ensues. In an attempt to identify the cellular source of IL-6 in Mdr2^−/−^ livers, we determined the expression of IL-6 in isolated hepatocytes and neutrophils from WT FVB and Mdr2^−/−^ mice, before and 40 h p.i (**Figure 5A**). Interestingly, there is no significant differences in IL-6 expression in isolated hepatocytes from WT FVB and Mdr2^−/−^ before and 40 h p.i. (**Figure 5A**), suggesting that hepatocytes did not contribute to the upregulation of IL-6 observed in Mdr2^−/−^ mice during infection. In contrast, we observed a significant increase of IL-6 expression in neutrophils isolated from Mdr2^−/−^ bone marrow before and 40 h p.i. compared to WT FVB neutrophils (**Figure 5A**). In these experiments, neutrophils from the bone marrow were used because of the paucity of neutrophils isolated from the livers which did not allow us to perform exploitable RT-PCR analysis. This result strongly suggests that IL-6^+^ neutrophils play a critical role in controlling parasite development in the liver of Mdr2^−/−^ mice.

To test the relevance of IL-6 in the elimination of the parasite in the liver of infected Mdr2^−/−^ mice, we compared the parasite development in WT (FVB) and WT (C57BL/6) mice to Mdr2^−/−^ (C57BL/6) and Mdr2 IL-6 (C57BL/6) double-knockout (Mdr2^−/−^IL-6^−/−^) mice. As shown in **Figure 5B**, while the parasite develops perfectly well in WT C57BL/6 mice (open circles), there was a strongly reduced development of the parasite in the liver of C57BL/6 Mdr2^−/−^ mice (open squares), a phenotype that is slightly less pronounced than that observed in WT FVB Mdr2^−/−^ mice (filled squares). More interestingly, infection phenotype of Mdr2^−/−^IL-6^−/−^ mice (filled triangles) was partly reversed and parasitemia tended to reach that of WT C57BL/6 mice (open circles). To highlight the differences in parasitemia between different groups of mice, results were expressed in log scale as it appears in the insert of **Figure 5B**. This reversal in the infection phenotype consecutive to IL-6 deficiency clearly demonstrates that the impaired parasite development in the liver of Mdr2^−/−^ mice is directly associated with the up-regulation of IL-6 production and strongly suggests that IL-6 represents a key anti-parasite cytokine in these mice.

### Abrogation of Parasite Liver Stage Development Upon Treatment With Recombinant IL-6 Which Recapitulates the Entire Phenotype of Mdr2^−/−^ Mice With Regard to the Control of Parasite Infection

To provide direct evidence that IL-6 could recapitulate the strong pro-inflammatory response induced by LPS (data not shown), WT WT FVB mice received recombinant murine IL-6 (rmIL-6) at doses of 1, 5 and 10 μg (i.p) administered one day before, the same day and two consecutive days after SPZ inoculation. Parasitemia was inhibited in a dose-dependent manner with a complete abrogation at 10 μg/mouse (**Figure 5C**). Survival curves showed similar patterns as parasitemia with a 40% survival at a dose of 5 μg/mouse and 100% survival at a dose of 10 μg/mouse (**Figure 5D**). These data demonstrate that elicitation of IL-6 early during liver infection completely abrogates parasite growth.

**Figure 6:**
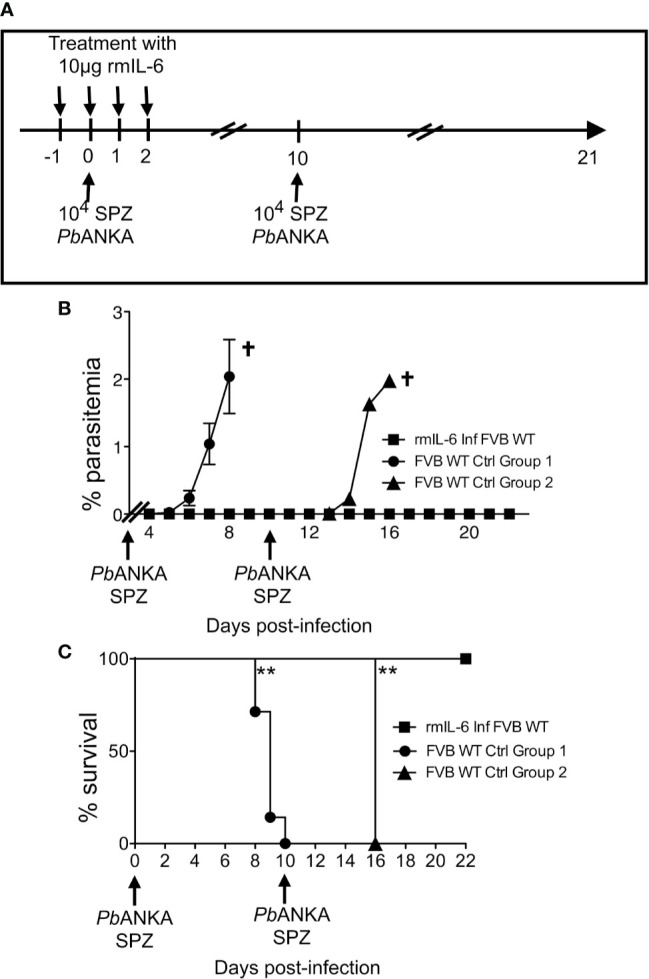
IL-6 alone recapitulates the entire phenotype of Mdr2^−/−^ mice with regard to the control of parasite infection. **(A)** Schematic representation of the experimental procedure. Six-week-old WT FVB mice (n = 6) were treated with recombinant murine IL-6 (rmIL-6) and infected with 10^4^ *Pb*ANKA SPZs. The same mice were challenged with 10^4^ and 5 × 10^4^ *Pb*ANKA SPZs at days 10 and 18 p.i. Non-treated six week-old WT FVB mice (n = 6 per group) were infected and used as controls for D10 and D18 challenges. Parasitemia **(B)** and Survival rate was recorded over time (Kaplan–Meier survival rates, Mantel–Cox test **0.0013 < p < 0.0053) **(C)**. Data are representative of three independent experiments.

Since IL-6 was found to represent the key mechanism by which Mdr2^−/−^ mice control the parasite infection, we postulated that treatment with IL-6 when mice are inoculated with *Pb*ANKA SPZs would not only prevent primary infections but also promote the development of a long-lasting anti-parasite protection. WT FVB mice were treated with 10 μg IL-6 one day before, the same day, and two consecutive days after inoculation of 10^4^ SPZs (**Figure 6A**). Mice which did not receive IL-6 developed parasitemia (**Figure 6B**) and died from ECM (**Figure 6C**, control group 1), whereas IL-6-treated mice did not show any parasitemia and all survived ECM (**Figures 6B, C** **)**. Protected mice, challenged at day 10 with the same dose of SPZs (**Figure 6A**) did not develop any parasitemia (**Figure 6B**) and all survived, whereas control mice developed parasitemia and all died from ECM at day 16 (**Figures 6B, C**, control group 2). The same mice which survived after two SPZ infection episodes were challenged at day 18 with a higher dose of 5 × 10^4^ SPZs. While all mice infected with this large dose developed parasitemia and died, repeatedly infected IL-6 treated mice remained parasite free. These data demonstrate that the presence of IL-6 during the pre-erythrocytic phase is able to protect mice from infection, possibly conferring a long-lasting anti-parasite immunity.

### Inoculation of SPZ in Mdr2^−/−^ Mice Results in a Robust T Cell Memory and Antibody Responses

In addition to neutrophils which represent a first barrier of defense against infections, we decided to investigate whether the abortive development of SPZ into blood stage parasites in MDR2^−/−^ mice will result in adaptive immune protection against repetitive and higher doses of inoculated SPZ. A protocol of immunization shown in **Supplementary Results: Figure 3A** represents four groups of mice: naïve mice, primed mice which received one single dose of 10^4^ SPZ and two challenged groups of mice which received two additional injections of 10^4^ (group 1) and 10^5^ SPZ (group 2), respectively, at 10 days interval. Parasitemia (**Supplementary Results: Figure 3B**) and survival rates (**Supplementary Results: Figure 3C**) were determined over time, and 3 months later, mice were sacrificed, and anti-SPZ antibodies were measured (**Supplementary Results: Figure 3D**). It appears that, except from occasional breakthroughs toward blood stage (**Supplementary Results: Figure 3B**), development in very few numbers of mice could be observed but ultimately cleared the parasite. The infected mice remained refractory to infection even at challenges with the high dose of 10^5^ SPZ. At the end of the experiment, 90 days after the initial infection, measurement of anti-SPZ specific IgG antibodies indicated that these remained at a high level and were elicited in a dose dependent fashion (**Supplementary Results: Figure 3D**).

**Figure 7:**
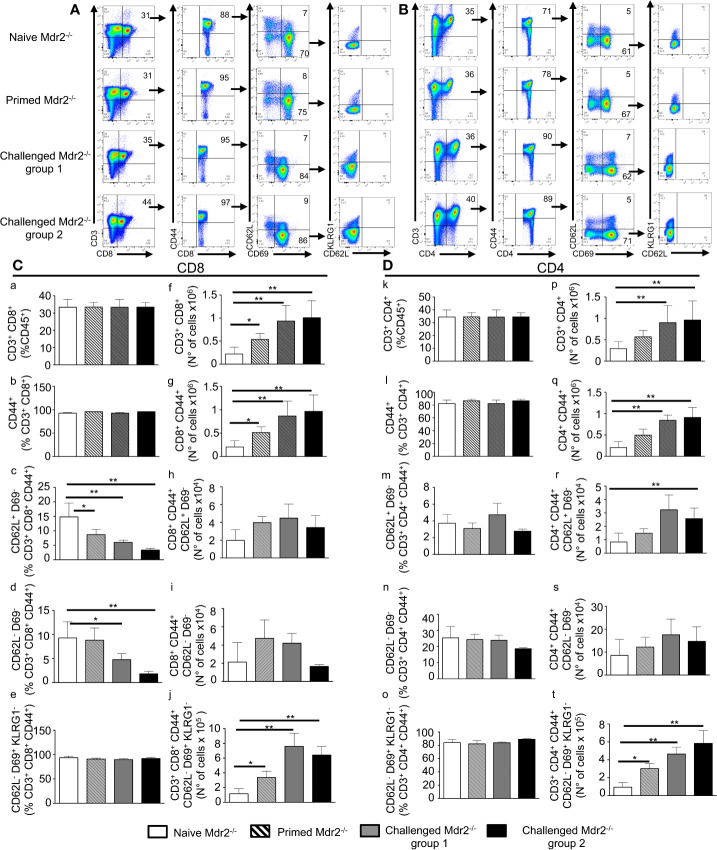
Inoculation of SPZ in Mdr2^−/−^ mice results in a CD8^+^ and CD4^+^ effector, central, and tissue resident memory T cell expansion. Six-week-old Female Mdr2**^−/−^** naïve mice, primed mice which received one single dose of 10^4^ SPZ, and two challenged groups of mice which received two additional injections of 10^4^ (group 1) and 10^5^ SPZ (group 2) respectively, at 10 days interval (same protocol as in **Supplementary Results: Figure 3**), were sacrificed 90 days following the initial infection with *Pb*ANKA parasites (n = 6 per group). Among total liver leukocytes, CD8^+^ and CD4^+^ effector and central memory T cells were stained with the following markers: CD45, CD3, CD8, CD44, CD69, CD62L, and KLRG1. **(A)** Gating strategy for CD8 Tem (CD3^+^ CD8^+^ CD44^hi^ CD69^−^ CD62L^−^), Tcm (CD3^+^ CD8^+^ CD44^hi^ CD69^−^ CD62L^+^), and Trm (CD3^+^ CD8^+^ CD44^hi^ CD69^+^ CD62L^−^ KLRG1^−^) among CD45^+^ cells. **(B)** Gating strategy for CD4 Tem (CD3^+^ CD4^+^ CD44^hi^ CD69^−^ CD62L^−^) Tcm (CD3^+^ CD4^+^ CD44^hi^ CD69^−^ CD62L^+^), and Trm (CD3^+^ CD4^+^ CD44^hi^ CD69^+^ CD62L^−^ KLRG1^-^) among CD45^+^ cells. Counts of CD3^+^ CD8^+^ cells **(C)** and CD3^+^ CD4^+^ cells **(D)** analyzed by flow cytometry expressed as percentage (c–e, and k–o) of CD45^+^ cells and in absolute number of cells (f–j, and p–t). Counts of CD8^+^ CD44^hi^ cells **(C)** and CD4^+^ CD44^hi^ cells **(D)**, analyzed by flow cytometry, were expressed as percentage **(C b, D i)** and absolute number of cells **(C g, D q)** of CD3^+^ CD8^+^ or CD3^+^ CD4^+^, respectively. Counts of CD69^−^ CD62L^−^, and CD69^−^ CD62L^+^ cells analyzed by flow cytometry expressed as percentage (c, d, and m, n) and absolute number (h, i, and r, s) of cells of CD3^+^ CD8^+^ CD44^hi^ cells **(C)** or CD3^+^ CD4^+^ CD44^hi^ **(D)**. Counts of CD69^+^ CD62L^−^ KLRG1^-^ cells analyzed by flow cytometry expressed in percentage (e, o) and absolute number (j, t) of cells of CD3^+^ CD8^+^ CD44^hi^ **(C)** or CD3^+^ CD4^+^ CD44^hi^ cells **(D)**. The asterisks indicate significant differences using the Mann–Whitney test (*p < 0.05; **p < 0.01). Shown data are representative of three independent experiments.

In parallel, we examined whether this immunization protocol resulted in an increased number of antigen specific T cells with effector function which, in addition to antibodies, could act to clear the infection. It is well established that about 90% of the effector cells die during the 1–2 week long contraction phase, leaving a residual population of long-lived effector and central memory T cells (25, 26). The total number of CD3^+^ CD8^+^ and CD3^+^ CD4^+^ T cells gated on CD45^+^ cells (**Figures 7A, B** **)** was found to be increased mostly in challenged Mdr2^−/−^ mice with no difference between 10^4^ and 10^5^ SPZ doses (**Figures 7Cf, Dp**). One of the changes in the cell surface following T cell activation consists of the upregulation of CD44. Gating of the CD3^+^CD8^+^ T cell population on the CD44 activation marker (**Figure 7A**) revealed an increased absolute number of CD8^+^ (**Figure 7Cg**) and CD4^+^ T cells (**Figure 7Dq**) in Mdr2^−/−^ challenged mice as compared to naïve Mdr2^−/−^ mice, again with no significant difference between 10^4^ and 10^5^ SPZ doses. A gating strategy based on the expression of CD44, CD69, and CD62L cell surface markers resulted in two distinct CD4 and CD8 T cell populations, namely CD44^+^ CD69^−^ CD62L^+^ believed to be central memory T cells (Tcm) and CD44^+^ CD69^−^ CD62L^−^ identified as effector memory T cells (Tem). Challenged mice showed that although percentage of CD8^+^ Tcm (**Figure 7Cc**) and CD8^+^ Tem (**Figure 7Cd**) cell populations decreased as compared to control mice, there was no significant difference in absolute cell numbers (**Figures 7Ch, i**). Analysis of memory T cells among CD4^+^ T cells showed that only CD4^+^ Tcm cell population (**Figure 7Dr**) expanded in challenged Mdr2^−/−^ mice. A gating strategy, based on the expression of CD44, CD69, CD62L, and KLRG1 (**Figures 7A, B** **)**, revealed a distinct CD8^+^ T cell population CD44^hi^ CD69^+^ CD62L^−^ KLRG1^−^, believed to be liver-resident memory T cells (Trm). Analysis of Trm cells showed that in infected Mdr2^−/−^ mice which received one dose of 10,000 SPZ (primed mice group), we observed an expansion of CD8^+^ (**Figure 7Cj**) and CD4^+^ (**Figure 7Dt**) Trm cell populations, in terms of absolute cell number, significantly higher than in control mice. Interestingly, in challenged Mdr2^−/−^ groups where mice received two additional doses of 10,000 SPZ (group 1) and 50,000 SPZ (group 2), we observed a dramatic expansion in terms of absolute number of CD8^+^ (**Figure 7Cj**) and CD4^+^ (**Figure 7Dt**) Trm cell populations.

These data suggest that although EEFs in infected Mdr2^−/−^ mice do not persist more than 24 h in the liver, a priming of T cells nonetheless occurred, giving rise to populations of CD8^+^ and CD4^+^ Tem, Tcm, and Trm cells in parallel with a potent anti-SPZ antibody response. These results are also consistent with the idea that the creation of an inflammatory environment in the liver is deleterious to the parasite and creates the conditions for the establishment of an anti-parasite immune memory.

## Discussion

In this study, we demonstrate a clear phenotypic difference in the *Plasmodium berghei* parasite development between age and sex matched Mdr2^−/−^ mice and WT mice, and this was replicated with the same mutation in two genetic backgrounds, WT FVB and C57BL/6. This is in agreement with our hypothesis that liver inflammation potentially blocks the development of the *Plasmodium* liver stage by shifting the immunologically tolerant environment in the liver towards an effective anti-parasite immunity.

Constantly challenged by gut-derived pro-inflammatory stimuli, a set of mechanisms has been developed in the liver to ensure a state of tolerance. Liver hematopoietic cells as well as parenchymal cells contribute to the establishment of this tolerogenic environment. Both in humans and in mice, intrahepatic dendritic cells (DCs) exhibit unique properties distinct from those of extrahepatic DCs, as they display an immature phenotype with low expression level of co-stimulatory and MHC II molecules, reduced endocytic activity and production of anti-inflammatory prostaglandin E2 (27). As a result, intrahepatic DCs are poor stimulators of T cells (28) *via* the production of IL-10 and induction of regulatory T cells (10, 29). During malaria infection, when inoculated SPZs reach the liver, additional mechanisms are initiated including the increased up-regulation by the abundant circumsporozoite protein of cyclic AMP in traversed Kupffer cells (KC), which in turn inhibits the assembly of the NADPH oxidase (30). This results in the blockade of reactive oxygen species, a potent innate defense mechanism required to kill the parasite (30). Traversal of KC by SPZs, in addition to blocking the respiratory burst, was also shown to generate an anti-inflammatory cytokine profile (31). In another study, exposure of KC to infectious SPZ down-modulated MHC class I expression, inhibited IL-12p40 secretion, and resulted in reduced antigen presenting function (32). Disruption of the liver homeostasis would create the conditions that lead to the reversal of the tolerogenic conditions and the killing of the parasite.

In this study, we addressed this possibility by using a mouse model where a genetic defect in the *Mdr2* gene creates inflammatory conditions in the liver. In support of our hypothesis, in contrast to WT FVB mice, injected SPZ were blocked early in their development in the liver of Mdr2^−/−^ mice. Over the years, Mdr2^−/−^ mice models were extensively studied as they provided a tool for studying cholestasis and hepatocellular carcinoma, we first suspected an intrinsic defect of hepatocytes to support parasite development. This turned out not to be the case, since *ex vivo* cultures of primary hepatocytes from Mdr2^−/−^ and WT FVB mice in the presence of SPZ generated equivalent EEF counts. We then ascribed defect in the parasite development in Mdr2^−/−^ mice to a possible control by the pro-inflammatory tissue environment. The phenotypic manifestations of the Mdr2^−/−^ mutation are strain-dependent. Initially, the mutation was introduced in the WT FVB mouse strain (Mdr2^−/−^ FVB), and more recently this mutation was transferred into the C57BL/6 genetic background (33) and this study demonstrated significantly retarded hepatocellular carcinoma development and inhibition of chronic hepatitis between 2 and 3 months of age in Mdr2^−/−^ C57BL/6 mice. Accordingly, the more pronounced inflammatory response in Mdr2^−/−^ FVB mice was correlated with higher infiltration of immune cells including neutrophils, T cells, and macrophages (33). Remarkably, a decreased expression of genes regulating lipid metabolism and reactive oxygen species was observed in Mdr2^−/−^ C57BL/6 mice, suggesting an attenuated pathological phenotype as compared to Mdr2^−/−^ FVB mice. The differences between the two Mdr2^−/−^ strains in the magnitude of liver inflammation which explain the differences in the behavior of the strains with respect to *Plasmodium* infection strongly support the concept we have put forward establishing the inter-relationship between liver inflammation and host resistance to *Plasmodium* infection.

Neutrophils and IL-6 were found to be among the most discriminative components between WT and Mdr2^−/−^ mice that are critical in controlling the parasite development. Neutrophils are essential for maintaining immune surveillance in homeostatic conditions and can be rapidly recruited in inflamed tissue under the control of several signals including CXCR2 and CXCL5. Neutrophil release from the bone marrow is promoted by increased expression of CXCR2 and its ligands (34) and IL-6 contributes to the increased mobilization and number in the periphery (35). In support of this, it has been reported that IL-6 controls neutrophil trafficking during inflammatory responses (36) and treatment with anti-IL-6 mAb suppressed neutrophilic inflammation (37). It is believed that IL-6 promotes neutrophil influx and survival by stimulating endothelial cells to secrete IL-8 which contributes to the recruitment of neutrophils at the site of inflammation (38) and possibly extending neutrophil half-life by preventing neutrophils from apoptosis (39). During inflammation, IL-6 was found to be constitutively stored by murine neutrophils (24, 40) and its secretion upon TLR ligands is the major inducer of the hepatic acute phase proteins (41). Related to this, we previously reported that during the pre-erythrocytic phase, a defect in *Plasmodium* parasite growth was caused by an early rise in IL-6 production by neutrophils (24). Also, work in rodents and cultured hepatocytes showed that inhibition of liver-stage development was mediated by IL-6 (42, 43). One of the proposed mechanisms by which IL-6 controls liver infection is by regulating iron homeostasis by hepcidin (44), which limits *Plasmodium* development inside hepatocytes (45).

Having access to an Mdr2^−/−^ and IL-6^−/−^ double knock out mice in the C57BL/6 background, we could address the relevance of increased IL-6 response in the defective parasite development in the liver of Mdr2^−/−^ mice. Evidence for the critical role of IL-6 in the acquisition by Mdr2^−/−^ mice of anti-parasite resistance was provided by the reversal of the infection phenotype from parasite blockade in Mdr2^−/−^ C57BL/6 mice to parasite development into blood stage in Mdr2^−/−^ IL-6^−/−^ double knockout mice. The current view that emerges from this model, is that the pre-existing pro-inflammatory environment in the liver of Mdr2^−/−^ mice becomes enriched in the IL-6 cytokine at the moment where the tissue receives *Plasmodium* SPZs and the blockade of parasite growth is very likely due to effector mechanisms associated with neutrophils. Neutrophils which respond to IL-6 signaling were also found to produce IL-6 (46), and this paracrine/autocrine loop may fuel the production of reactive oxygen species (ROS) which are known to be elevated in Mdr2^−/−^ mice (47) ultimately resulting in the parasite blockade. As a bridge between these innate immune signaling pathways and subsequent adaptive immune responses, IL-6 was shown to support the growth and enhancement of antibody production by B cells (48). In support of this, IL-6-deficient mice are impaired in their IgG production upon immunization (49). Although IL-6 has been tightly associated to B cell biology as shown in earlier studies, B cells were found to express only limited levels of IL-6 receptor compared to other leukocytes (50). Accordingly, IL-6 effects on B cells are due to its indirect effects on effector T cells, especially Tfh cells (50), and perhaps other T cell subsets.

IL-6 is critical in regulating CD4 T cell differentiation and effector cytokine production by promoting IL-4 production during T cell activation and IL-21 production, an essential effector cytokine produced by Tfh cells (51, 52). In earlier studies, IL-6 in combination with IL-7 signaling promotes CD8 memory T cell generation after vaccination (53).

An important question is whether the rapid clearance of SPZ resulted in the priming of immune cells such as CD8^+^ T cells which were previously shown to correlate with protection against challenge with *P. berghei* in mice (54) and against irradiation-attenuated *P. falciparum* SPZ immunization in humans (55). A subset of CD8^+^ T cells, called tissue-resident memory T cells (Trm), has been identified as sentinels against invading pathogens, in particular they are capable of recognizing infected hepatocytes, and their depletion abrogated protection in mice (56, 57). In the present report, we looked whether, due to a pro-inflammatory environment in the liver, CD8^+^ and CD4^+^ Trm cells are enhanced upon SPZ vaccination in Mdr2^−/−^ mice. Our results show that not only total CD8^+^ and CD4^+^ cells but also CD8^+^ and CD4^+^ Trm cells were present at a significantly higher levels in SPZ challenged Mdr2^−/−^ mice as compared to control non challenged mice. Repeated introduction of SPZ in this inflamed tissue environment dramatically increased and maintained over a significant amount of time the number of these cell populations which corresponds to the establishment of an immune memory.

Although not directly demonstrated in the present work, it is possible that the inflammatory events associated with neutrophil-mediated innate immunity may shape the function of differentiated effector and memory CD4 and CD8 T cells. Conversely, CD4 and CD8 T cells can promote acute inflammation by secreting IFN-γ and TNF-α which allows the attraction of neutrophils *via* the up-regulation of endothelial adhesion molecules. Interestingly, neutrophils were shown to pick up and transport antigens to the lymph nodes for CD8 T cell priming (58) and to the bone marrow for the maintenance of memory CD8 T cells (59). Thus, neutrophils not only initiate rapid anti-parasite innate immune responses, but also determine the specificity and the magnitude of the CD8 T cell responses as evidenced by a previously unappreciated role for neutrophils as substrates for cross-priming of CD8 T cell responses against bacterial antigens (60). Similarly, it was tempting to associate the early IL-6 signaling in the liver with the parasite clearance and the subsequent elicitation and expansion of CD4 and CD8^+^ cells under the control of IL-6 shown to protect against infections caused by several pathogens. The failure of parasite development in Mdr2^−/−^ mice which are completely refractory to infection even after multiple challenges with high doses of SPZ suggests that generation of T cells upon a successful vaccination regimen might have taken place in these mice. To this end, we examined the generation of CD4 and CD8 effector memory (Tem) and central memory (Tcm), defined on the basis of the surface expression of CD44, CD69, and CD62L after a long period of priming and challenges of Mdr2^−/−^ mice with SPZ. These two subsets of memory T cells were previously reported in an alternative immunization protocol whereby following radiation-attenuated SPZ immunization, the overall population of intrahepatic CD8 T cells significantly increases as compared to naïve mice and protection was linked to both increased CD8 Tem and Tcm (61). In our model, it was striking to observe that 70 days after the last SPZ challenge, in contrast to radiation-attenuated SPZ immunization protocol, challenged MDR2^−/−^ mice only maintained a substantially elevated absolute numbers of CD4 Tcm cells, but not Tcm CD8 cells nor CD8 and CD4 Tem cells. This is also in contrast to *P. chabaudi*-infected mice that continue to generate Tem long time after parasite clearance (62). The discrepancies between these findings are very likely due to the particular genotype of mice used in our study and also to the particular inflammatory events taking place in Mdr2^−/−^ mice.

According to our data, it is tempting to speculate about a possible impact of contemporaneous liver inflammatory episodes on malaria transmission and disease progression in individuals living in endemic areas. In this context, hepatitis B virus (HBV) infections are very common in many of the malaria endemic regions. A study in Gambian children showed that impaired clearance of liver stage parasites may relate to the reduced level of HLA class I antigen expression on HBV-infected hepatocytes (63). We believe their interpretation is not valid since loss of HLA class I antigens would lead to hepatocyte killing by NK cells. Instead, we suggest that most probably the nature of the inflammatory response in these infected children may not be appropriately adjusted to break tolerance and hence parasite clearance. In another study conducted in the Brasilian Amazon, *Plasmodium* parasitemia inversely correlated with HBV viremia, and HBV infections diminish the intensity of malaria episodes, and this holds true for both *P. falciparum* and *P. vivax* infections (64). These results were supported by the work of Dabo et al. (65) who showed reduced parasitemia among subjects with HBV and malaria co-infection. This is in contrast to another report (66) which showed that the presence of HBV could significantly negatively affect the prognosis of malaria infection. The inconsistencies observed in these human studies may be related to various limitations, including the lack of available immunological parameters, and no screen for helminth infections nor genetic deficiency such as glucose-6 phosphate dehydrogenase (G6PD) were undertaken.

In conclusion, the introduction for the first time of Mdr2^−/−^ mice as a mouse model for malaria infection allowed us to validate the concept that the shift of the naturally tolerant environment in the liver towards a pro-inflammatory state promotes an anti-parasite killing and the establishment of an effective immune memory. This model can be instrumental in designing new approaches for malaria prevention such as the development of effective vaccines against pre-erythrocytic stages of the parasite. In particular, the central position of IL-6 in determining long term anti-parasite immunity makes feasible the idea of ​​using the delivery of this cytokine or its up-regulation by an appropriate means concomitantly with parasite inoculation as credible vaccine approaches.

## Data Availability Statement

The raw data supporting the conclusions of this article will be made available by the authors, without undue reservation.

## Ethics Statement

The animal study was reviewed and approved by Animal studies approved by the “comité d’éthique en expérimentation animale” (CETEA) (Permit Number N° dap180040 issued on 2018).

## Author Contributions

Conceived the project: SM. Writing the original draft: SM, MG. Writing, review and editing: JA, RC, VH, RA, SM, CD-G. Methodology: RC, VH, CD-G, RA, YW, JA. Designed the experiments: SM, MG. Performed the experiments: MG, MW, CD-G, RP, P-HC, AP. All authors contributed to the article and approved the submitted version.

## Funding

This work has been supported by the French Parasitology consortium ParaFrap (ANR-11-LABX0024), and by a grant from Institut Pasteur to MG, and SM. CD-G was supported by a postdoctoral fellowship from the Helmut Horten Foundation, Agno, Switzerland.

## Conflict of Interest

The authors declare that the research was conducted in the absence of any commercial or financial relationships that could be construed as a potential conflict of interest.
