# Supplementary figures and images for: Hepatic Inflammation Confers Protective Immunity Against Liver Stages of Malaria Parasite
Source: Front Immunol. 2020 Nov 19;11:585502. doi: 10.3389/fimmu.2020.585502 (PMC7710885; doi:10.3389/fimmu.2020.585502)

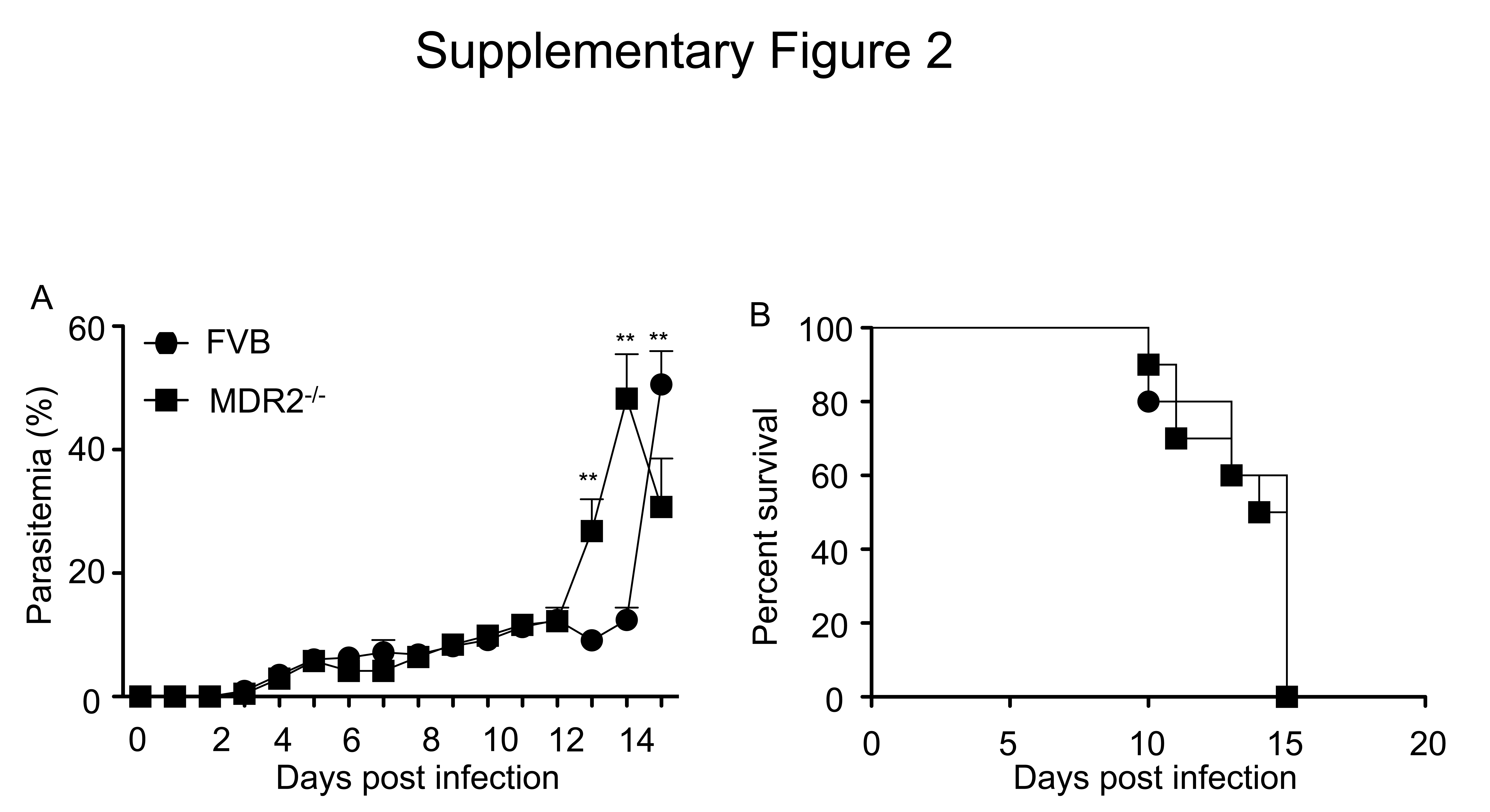

Supplement: Supplementary file 2 [file Image_2.tif]

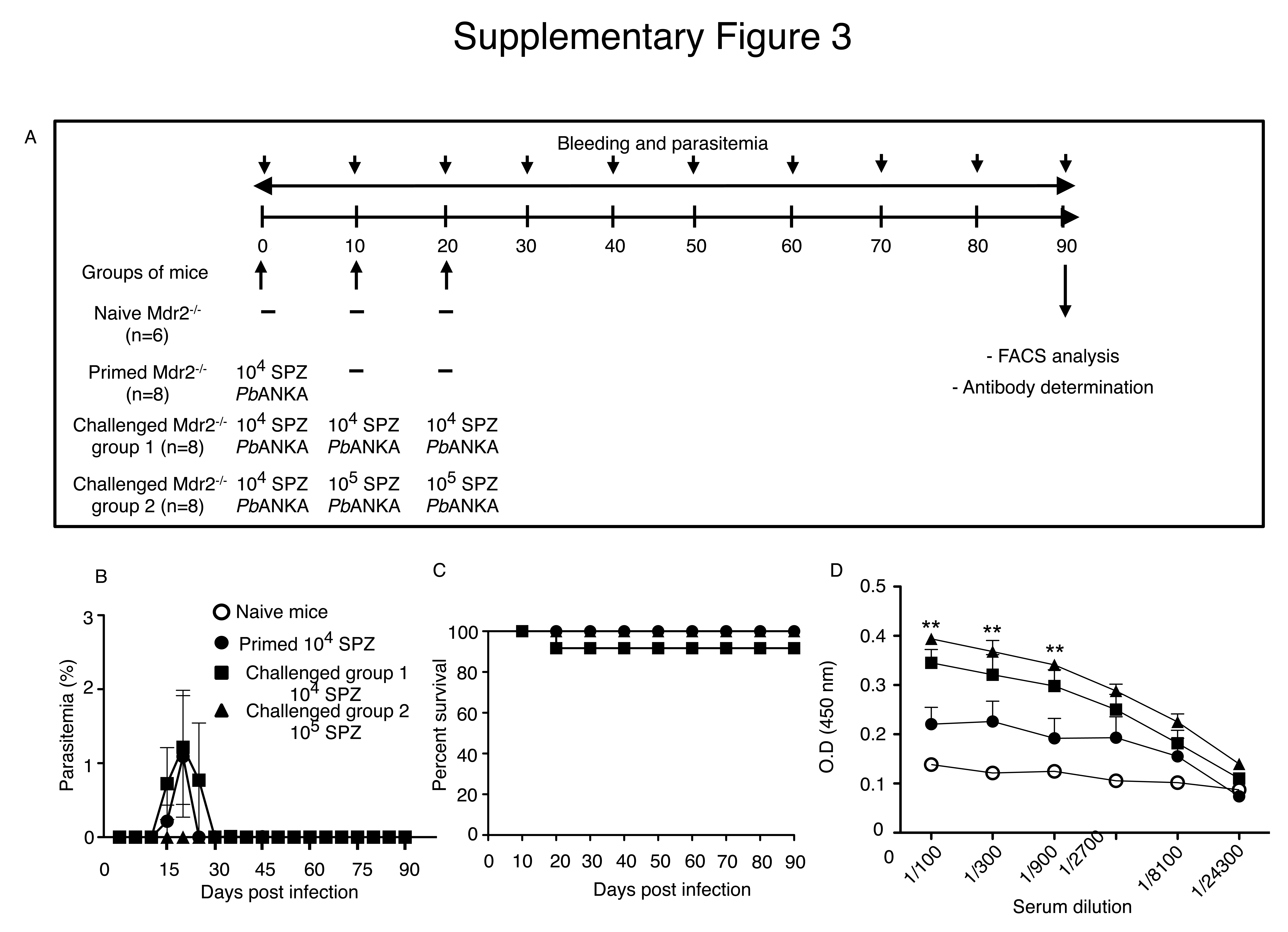

Supplement: Supplementary file 3 [file Image_3.tif]
