# Supplementary material for: Hepatic Inflammation Confers Protective Immunity Against Liver Stages of Malaria Parasite
Source: Front Immunol. 2020 Nov 19;11:585502. doi: 10.3389/fimmu.2020.585502 (PMC7710885; doi:10.3389/fimmu.2020.585502)
Supplement: Supplementary file 4 [file Table_1.docx]

**Supplementary Material**

**Supplementary results: Fig. 1: Parasitemia and survival of infected FVB and Mdr2^-/-^** **with *P.berghi*.**

Infection with *Pb*ANKA (A-F). Six-week-old FVB and Mdr2^-/-^ mice (n=6 per group) were infected i.v. with 10^2^ (A, B,), 10^3^ (C, D) or 10^4^ (E, F) SPZ of *Pb*ANKA. Kaplan-Meier survival plots (Mantel-Cox **p<0.047 *p=0.019) and parasitemia were recorded over time. Infection with *Pb*NK65 (G-L). Six-week-old WT FVB and Mdr2^-/-^ mice (n=6 per group) were infected i.v. with 10^2^ (G, H), 10^3^ (I, J) or 10^4^ (K, L) SPZ of *Pb*NK65. Kaplan-Meier survival rates (Mantel-Cox ***p=0.0006) and parasitemia were recorded over time. Results are representative of two independent experiments.

**Supplementary results: Fig. 2: in contrast to SPZ infection, Mdr2^-/-^ mice support parasite development when inoculated with blood stage parasites**

Six-week-old FVB and Mdr2^-/-^ mice (n=6 per group) were infected i.v. with 10^5^ *Pb*ANKA iRBCs. Parasitemia (A) and survival rates (B) were recorded over time. The asterisks indicate that significant differences invariably exist between Mdr2^-/-^ and WT FVB groups using the Mann Whitney test (**, 0.004 < P < 0.01). Data are representative of two independent experiments.

**Supplementary results: Fig. 3:** **Inoculation of SPZ In Mdr2^-/-^ mice results in a robust antibody response**. (A) Schematic representation of the experimental procedure. Six week-old female Mdr2^-/-^mice were distributed into 4 groups (n= 6 per group): naïve mice, primed mice which received a single dose of 10^4^ SPZ and 2 challenged groups of mice which received two additional injections of 10^4^ (group 1) and 10^5^ SPZ (group 2), respectively, at 10 days interval. Parasitemia (B) and Kaplan-Meier survival rates (C) (Mantel-Cox test **0.0013<p<0.0053) were recorded over time. (D) Measurement by ELISA of antiparasite-specific IgG antibodies in protected Mdr2^-/-^ mouse sera at day 90 p.i. with *Pb*ANKA parasites. Data are representative of two independent experiments. **, 0.0079 < P < 0.01; Mann-Whitney test.

| **Primer** | **Fw/Rev** | **Sequence** |
| --- | --- | --- |
| *Pb* 18S | Fw  Rev | ATTAATCTTGAACGAGGAATGGCT  TCAATCGGTAGGAGCGACG |
| mu HPRT | Fw  Rev | CTGGTGAAAAGGACCTCTCG  TGAAGTACTCATTATAGTCAAGGGCA |
| mu IL-6 | Fw  Rev | AAAGAAATGATGGATGCTACCAAAC  CTTGTTATCTTTTAAGTTGTTCTTCATGTACTC |
| mu CXCL5 | Fw  Rev | GCATTTCTGTTGCTGTTCACGCTG  CCTCCTTCTGGTTTTTCAGTTTAGC |
| mu CXCR2 | Fw  Rev | ATGCCCTCTATTCTGCCAGAT  GTGCTCCGGTTGTATAAGATGAC |

**Table S1 : Sequences of primers**

Sequences of the forward and reverse primers used to detect and quantify the listed genes
